# Evaluation of Lymphocyte Response to the Induced Oxidative Stress in a Cohort of Ageing Subjects, including Semisupercentenarians and Their Offspring

**DOI:** 10.1155/2018/7109312

**Published:** 2018-02-19

**Authors:** Federico Sizzano, Sebastiano Collino, Ornella Cominetti, Daniela Monti, Paolo Garagnani, Rita Ostan, Chiara Pirazzini, Maria Giulia Bacalini, Daniela Mari, Giuseppe Passarino, Claudio Franceschi, Alessio Palini

**Affiliations:** ^1^Flow Cytometry, Nestlè Institute of Health Sciences, EPFL Campus, 1010 Lausanne, Switzerland; ^2^Molecular Biomarkers, Nestlè Institute of Health Sciences, EPFL Campus, 1010 Lausanne, Switzerland; ^3^Department of Experimental and Clinical Biomedical Sciences, University of Florence, Viale Morgagni 50, 50134 Florence, Italy; ^4^Department of Experimental, Diagnostic and Specialty Medicine, Experimental Pathology, University of Bologna, Via S. Giacomo 12, 40126 Bologna, Italy; ^5^Interdepartmental Centre “L. Galvani” (CIG), University of Bologna, Via S. Giacomo 12, 40126 Bologna, Italy; ^6^Center for Applied Biomedical Research, St. Orsola-Malpighi University Hospital, 40138 Bologna, Italy; ^7^Clinical Chemistry, Department of Laboratory Medicine, Karolinska Institute at Huddinge University Hospital, S-141 86 Stockholm, Sweden; ^8^Unit of Bologna, CNR Institute for Molecular Genetics, Bologna, Italy; ^9^Laboratory of Musculoskeletal Cell Biology, Rizzoli Orthopedic Institute, Bologna, Italy; ^10^IRCCS, Institute of Neurological Sciences of Bologna, Ospedale Bellaria, Via Altura 3, 40139 Bologna, Italy; ^11^Geriatric Unit, Department of Medical Sciences and Community Health, University of Milan, Via Pace 9, 20122 Milan, Italy; ^12^Fondazione Ca' Granda, IRCCS Ospedale Maggiore Policlinico, Via Francesco Sforza 35, 20122 Milan, Italy; ^13^Department of Ecology, Biology, and Earth Sciences, University of Calabria, Rende, Italy

## Abstract

The production of reactive oxygen species (ROS) may promote immunosenescence if not counterbalanced by the antioxidant systems. Cell membranes, proteins, and nucleic acids become the target of ROS and progressively lose their structure and functions. This process could lead to an impairment of the immune response. However, little is known about the capability of the immune cells of elderly individuals to dynamically counteract the oxidative stress. Here, the response of the main lymphocyte subsets to the induced oxidative stress in semisupercentenarians (CENT), their offspring (OFF), elderly controls (CTRL), and young individuals (YO) was analyzed using flow cytometry. The results showed that the ratio of the ROS levels between the induced and noninduced (I/NI) oxidative stress conditions was higher in CTRL and OFF than in CENT and YO, in almost all T, B, and NK subsets. Moreover, the ratio of reduced glutathione levels between I/NI conditions was higher in OFF and CENT compared to the other groups in almost all the subsets. Finally, we observed significant correlations between the response to the induced oxidative stress and the degree of methylation in specific genes on the oxidative stress pathway. Globally, these data suggest that the capability to buffer dynamic changes in the oxidative environment could be a hallmark of longevity in humans.

## 1. Introduction

Immunosenescence is characterized by age-associated changes in cell phenotype and function that ultimately leads to a general impairment of the immune response [1]. In the innate compartment, in mice as well as in humans, a decrease in neutrophil chemotaxis, phagocytosis, and oxidative burst has been observed along with a decrease in natural killer (NK) cells and macrophage cell functions [2, 3]. Changes in the acquired immunity during ageing are driven by the thymic involution, leading to a decreased production of naïve T cells capable of replenishing the peripheral pool [4]. Furthermore, homeostatic mechanisms as well as persistent infections (i.e., cytomegalovirus) push memory T cells towards several rounds of replication during the ageing process [5–7]. Once reached the replicative senescence, these cells show energy, resistance to apoptosis, and changes in cytokine production [8]. Moreover, the impairment of the immune function during the ageing process can be even promoted by its inability to restore a proper balance between prooxidant, such as reactive oxygen species (ROS), and antioxidant molecules, such as the enzymes superoxide dismutase and catalase or the reducing agent glutathione (GSH). This condition is commonly defined as oxidative stress [9, 10]. ROS are continuously produced in the mitochondria as a result of the reduction of a small percentage of molecular oxygen by leaked electrons in the electron transporting chain or are produced enzymatically by the NADPH oxidase, mediating the respiratory burst in phagocytes. Other sources of ROS include the xanthine oxidase or the nitric oxide synthase pathways as well as environmental ultraviolet or ionizing radiation [11]. ROS play an important role in the immune system other than the respiratory burst, participating in the T, B, NK, and dendritic cell signaling or coordinating the cytokine production [12–14]. Nevertheless, ROS mediate detrimental effects to different cellular components if not properly counterbalanced by the antioxidant system within the cell. Such effects include lipid peroxidation, DNA oxidative damage, and protein modifications like glutathionylation and carbonylation [15, 16]. As an example, in the immune system, it has been shown that the oxidative modifications of proteins can alter the tertiary structure of the self-molecules, thus representing a risk factor for the development of autoimmune reactions [17, 18]. Moreover, oxidative changes can impair the proteasome functions, resulting in an uncorrected processing of peptides for T cell presentation by MHC molecules [19]. Immunosenescence per se (thymic involution, memory T cell expansion, and replicative senescence) and oxidative stress are key stimuli for fueling the low grade, persistent, and systemic inflammatory status, known as “inflammaging” [20], that is one of the pillars of the ageing process and strongly contributes to the pathogenesis of the main age-related diseases in humans. In this framework, centenarians who have survived, escaped, or delayed the onset of major age-related diseases achieving the extreme limits of the human life-span appear to have reached a balanced status between the inflammaging and the so called *anti-inflammaging*; that is, the process, involving molecules and their related pathways, aimed to control and modulate the inflammation and/or the oxidative stress [21]. Data in literature show that centenarians have less amounts of plasma oxidative stress markers [22, 23] or increased amounts of antioxidant vitamins [24]. However, there is a lack of experimental data in human models on the oxidative stress measured directly in the cells, and especially in white blood cells, from centenarians. In fact, such determinations were performed using cells from elderly subjects who turned 95 yo as a maximum [25–27]. Moreover, these studies performed mainly the oxidative stress determination at the baseline, that is, without inducing the ROS production. As the organisms face dynamic changes of the oxidative environment [28], we questioned if the capability of a cell in resisting the induced oxidative stress could be a hallmark of an efficient *anti-inflammaging* and thus of healthy ageing and longevity. To the best of our knowledge, only limited data on the response to induced oxidative stress in centenarians is available in literature [29]. In this paper, in order to provide further evidence to this concept, we performed an *in-depth* cell immunophenotyping and oxidative stress analysis by means of polychromatic flow cytometry (PFC) in a cohort of semisupercentenarians (CENT), elderly controls (CTRL), and young (YO) individuals. Moreover, the present analysis includes cells from centenarians' offspring (OFF), individuals representing an informative model to identify trajectories of healthy aging and their determinants (genetic and environmental). PFC was used to analyze the response to induced oxidative stress as ROS and GSH relative fluorescence in the main subsets of T, B, and NK cells. The phenotypic changes were also evaluated by PFC in order to illustrate the ongoing process of immunosenescence in such cohort. DNA methylation profile of a list of genes involved in the response to oxidative stress was also analyzed, in order to determine whether changes in the ability to cope with oxidative stress may be related to epigenetic reasons.

## 2. Methods

### 2.1. Subject Cohort

The subjects described in this paper (CENT *n* = 7: 3 males (M)/4 females (F), median age/range 106 yrs/105–107 yrs; OFF *n* = 6: 3M/3F, median age/range 72 yrs/59–85 yrs; CTRL *n* = 7: 4M/3F, median age/range 72 yrs/59–77 yrs; and YO *n* = 7, 3M/4F, median age/range 35 yrs/25–37 yrs) were recruited all in Bologna and were part of a larger cohort described elsewhere [30]. The study protocol was approved by the Ethical Committee of Saint Orsola-Malpighi University Hospital (Bologna, Italy). Informed written consent for the participation was obtained at the time of blood withdrawal.

### 2.2. PBMC Separation

Diluted heparinized blood in PBS was carefully layered on Ficoll-Hypaque and centrifuged for 20 min at 600*g*. After removal of platelets by centrifuging for 10 min at 200*g*, PBMCs were resuspended in the frozen medium (FBS/10% DMSO), stored at −80°C for 24 hours and then transferred in liquid nitrogen until used.

### 2.3. Flow Cytometry

Cells were thawed in warm Iscove Modified Dulbecco's Medium (IMDM), centrifuged at 200*g* for 5 min, and resuspended in 100 *μ*l of IMDM. Briefly, PBMCs (2 tubes for T cell and 2 for B/NK detection) were incubated in the presence or absence of ROS inducer tert-butyl-hydroperoxide (TBHP) for 30 min at 37°C in 5% CO_2_, according to the manufacturer's instructions (Invitrogen, Carlsbad, CA, USA). Then, fluorescent probes for GSH (Thiol Tracker, TT, Invitrogen, ex: 405 nm, em: 525 nm) and ROS (CellROX green, Invitrogen, ex: 508 nm, em: 525 nm, specific for H_2_O_2_ and superoxide anion) were added at a final concentration of 5 *μ*M and 500 nM, respectively. Cells were incubated for additional 30 min at 37°C in 5% CO_2_. 50 *μ*l of mixtures of mAbs targeting the antigens for specific T, B, and NK subsets was then added to the respective tube for 15 min. Mix for T cells includes the following mAbs all purchased from Beckman Coulter (Bree, CA, USA): anti-CD25-phycoerhytrin (PE), CD45RO-PE-Texas Red, CD3-PE-cyanine 7 (Cy7), CD45-Alexa 700, and CD4-allophycocyanin- (APC-) Cy7. Anti-CCR7-peridinin chlorophyll-Cy5.5, CD127-Alexa 647, and anti-CD8-BUV395 were acquired from Becton Dickinson, (Franklin Lakes, NJ, USA). Mix for B and NK cells includes anti-CD16-PE, anti-CD38-PE-Texas Red, anti-CD27 PE-Cy5.5, anti-CD3-PC7, anti-CD56-APC, anti-CD45 APC-Alexa 700, and CD20-APC-Cy7 purchased from Beckman Coulter and CD19-BUV395 from Becton Dickinson. Samples were then diluted by adding 300 *μ*l of PBS and acquired by means of a LSORP Fortessa equipped with 5 laser lines (355 nm, 405 nm, 488 nm, 561 nm, and 640 nm) and 18 photomultipliers (PMTs). Dead cells were excluded using a viability dye (Live Dead, Invitrogen). All the reagents were titered according the internal SOPs. Moreover, to ensure consistent and reproducible results, we used the 8-peak Rainbow Beads (Spherotech, Lake Forest, IL, USA) as a fluorescent calibrator allowing us to trace the laser/PMT performance across the independent experimental runs. PBMC subsets were identified by a hierarchical gating strategy considering the shared (or not) CD antigens among the different populations. Data files were saved in the FCS 3.0 format and analyzed offline using the *Batch Analysis* function of the FCS Express 5.0 package (De Novo software, Glendale, CA, USA) allowing to export all percentages and fluorescence values in Excel format. The percentage of a given cell subset was referred to the parental gate (i.e., CD4 naïve in the CD4 gate or Tregs in CD4 memory gate). The level of ROS or GSH at the baseline was evaluated by the median fluorescence intensity (MFI) of the relative probe, whereas the response to induced oxidative stress was calculated as the ratio of MFIs of ROS (or GSH) between the I and NI conditions.

### 2.4. Methylation Profile

Whole-genome DNA methylation profile of PBMCs from the entire cohort has been described previously [30]. Briefly, genomic DNA was extracted from PBMCs with the AllPrep DNA/RNA/protein kit (QIAGEN, Hilden, Germany) and it was treated with sodium bisulphite using the EZ-96 DNA Methylation Kit (Zymo Research, Irvine, CA). Genome-wide DNA methylation level was assessed using the Infinium HumanMethylation450 BeadChip (Illumina, San Diego, CA) following the manufacturer's instructions, and the arrays were scanned by HiScan (Illumina).

### 2.5. Statistical Analysis

Differences among groups in T, B, and NK subsets for cell percentages or MFI of ROS and GSH were assayed using the nonparametric Kruskal-Wallis one-way analysis of variance followed by Dunn's posttest. Correlations between cell percentage and age or between ROS MFI ratios and the degree of methylation were assayed using Spearman correlation. Prism 5.0 was used for both analyses (GraphPad, La Jolla, CA, USA).

For DNA methylation analysis, beta values were extracted using minfi Bioconductor package. The differences among CENT, CTRL, and OFF were assessed by ANOVA with R software.

## 3. Results

### 3.1. Percentages of PBMC Subsets across the Age Groups

**Figure 1:**
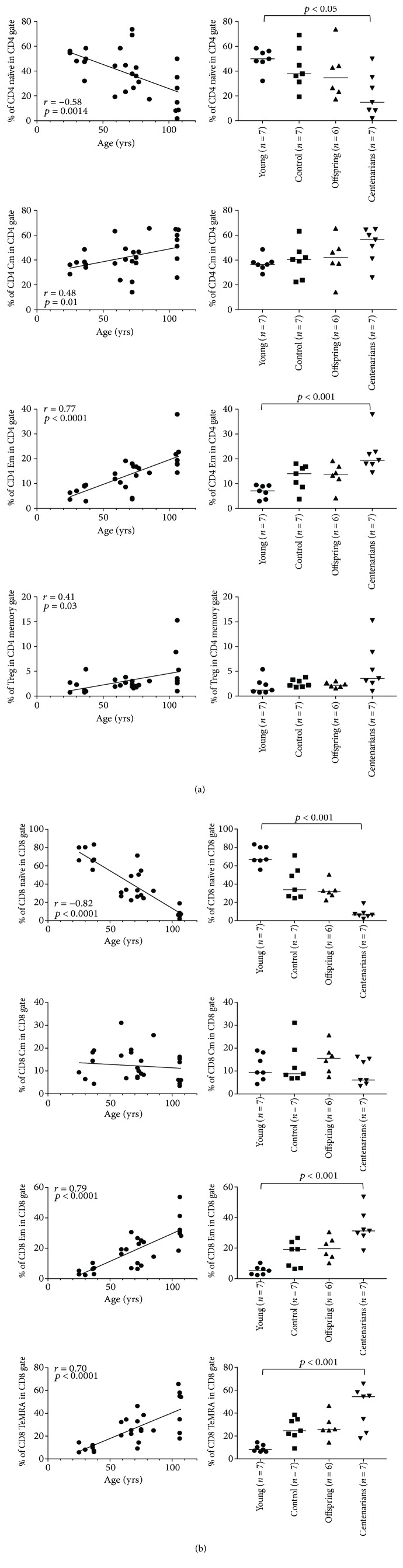
(a) Correlation between age and cell percentage and ANOVA for the cell percentage among the age groups in CD4 subsets. Overall ANOVA *p* values are as follows: CD4 naïve: *p* = 0.03; CD4 Cm: *p* = 0.14; CD4 Em: *p* = 0.0012; and Treg: *p* = 0.10. Significant results of Dunn's posttest are illustrated on the plots. (b) Correlation between age and cell percentage and ANOVA for the cell percentage among the age groups in CD8 subsets. Overall ANOVA *p* values for CD8 subsets are as follows: CD8 naïve: *p* < 0.0001; CD8 Cm: *p* = 0.28; CD8 Em: *p* = 0.0004; and CD8 TeMRA: *p* = 0.0014. Significant results of Dunn's posttest are illustrated on the plots.

**Figure 2:**
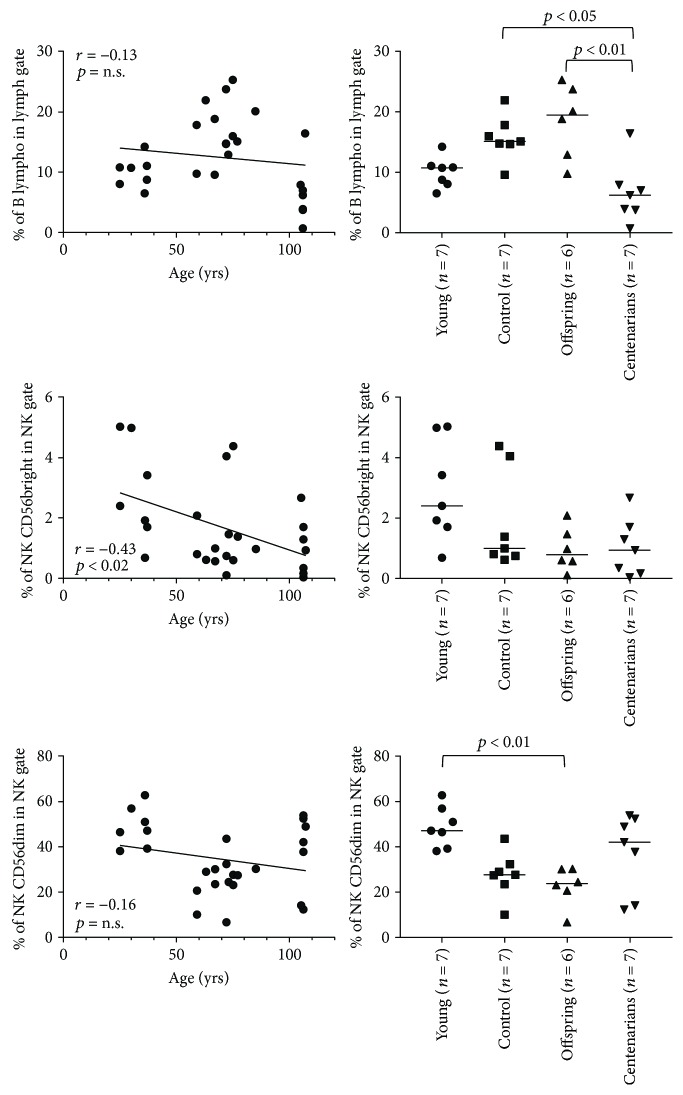
Correlation between age and percentage and ANOVA for the percentage among the age groups in B and NK subsets. Overall ANOVA *p* values for B cells: *p* = 0.002; NK CD56bright: *p* = 0.06; and NK CD56dim: *p* = 0.007. Significant results of Dunn's posttest are illustrated on the plots.

As expected, correlation analysis showed a significant age-related decrease in the percentage of naïve subsets both in CD4 and CD8 T lymphocytes (*p* = 0.0014 and *p* < 0.0001, resp.). Conversely, a significant age-related increase in CD4 central memory (Cm; *p* = 0.01), effector memory (Em; *p* < 0.0001), and regulatory T cells (Tregs; *p* = 0.03) as well as in CD8 Em and terminally differentiated effector memory (TeMRA) cells was observed (*p* < 0.0001; Figures 1(a) and 1(b)). Analysis of variance (ANOVA) showed significant differences in the percentages of CD4 and CD8 subsets (except for CD4 and CD8 Cm and Tregs) between YO and CENT. No differences between OFF and CTRL in the different subsets were appreciated (Figures 1(a) and 1(b)). When considering B and NK cells, correlation analysis showed a significant inverse correlation with the age of the CD56bright subset, whereas no age correlation in B or NK CD56dim cells was observed (Figure 2). ANOVA showed statistically significant differences in B and NK CD56dim percentages across the age groups. Percentages of B cells in CTRL and OFF were significantly higher than those in CENT. Notably, CENT showed similar percentages of B lymphocytes compared with YO. Lastly, CTRL and OFF showed lower percentages of NK CD56dim cells compared with YO and CENT. Likewise, B cells, CENT and YO showed similar percentages of NK CD56dim (Figure 2).

### 3.2. Oxidative Stress and GSH Content at the Baseline in PBMC Subsets

**Figure 3:**
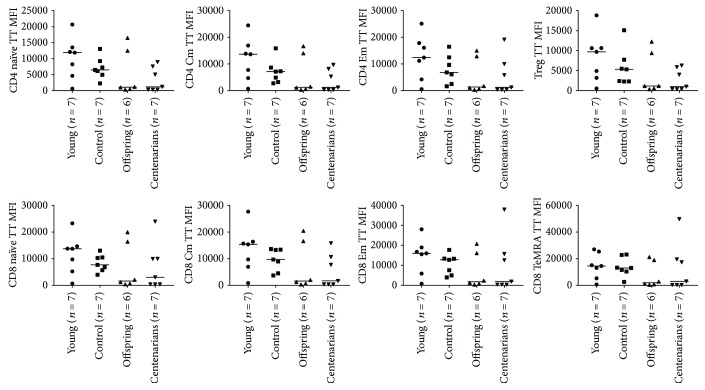
ANOVA for the levels of GSH expressed as MFI of the specific probe (Thiol Tracker, TT) among the age groups in CD4 and CD8 subsets. Overall ANOVA *p* values for CD4 subsets are as follows: CD4 naïve: *p* = 0.13; CD4 Cm: *p* = 0.16; CD4 Em: *p* = 0.20; and Treg: *p* = 0.15. Overall ANOVA *p* values for CD8 subsets are as follows: CD8 naïve: *p* = 0.38; CD8 Cm: *p* = 0.23; CD8 Em: *p* = 0.28; and CD8 TeMRA: *p* = 0.50.

**Figure 4:**
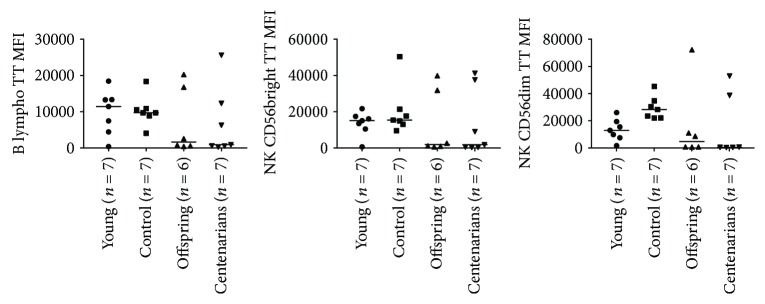
ANOVA for the levels of GSH expressed as MFI of the specific probe (Thiol Tracker, TT) among the age groups in B and NK subsets. Overall ANOVA *p* values for B cells: *p* = 0.50; NK CD56bright: *p* = 0.35; and NK CD56dim: *p* = 0.07.

The levels of ROS and GSH in noninduced (NI) condition were determined by means of the fluorescent probes. No significant differences among the age groups were observed, although a tendency towards higher levels of ROS in the YO was observed in several subsets (see Table S2). Results of GSH analysis showed a lower GSH level in OFF and CENT compared with YO and CTRL in all PBMC subsets considered, although ANOVA did not show significant differences among the age-groups (Figures 3 and 4 and Table S4).

### 3.3. Response of the PBMC Subsets to Induced Oxidative Stress

**Figure 5:**
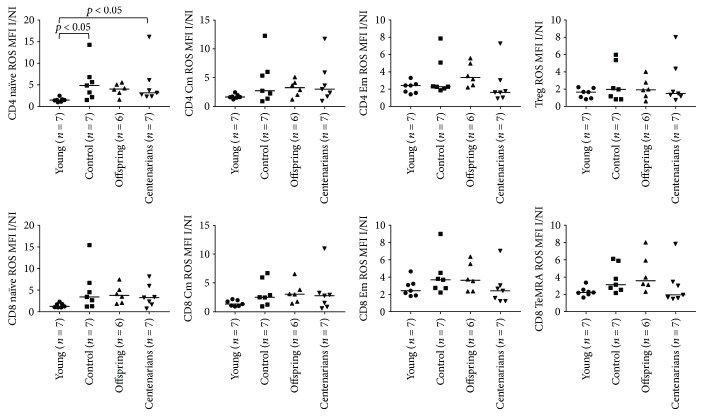
ANOVA for the ratios of the levels of ROS (expressed as MFI values of the ROS probe) between the I and NI oxidative stress conditions in CD4 and CD8 subsets among the age groups. See Table 1 for overall ANOVA *p* values. Significant results of Dunn's posttest are illustrated on the plots.

**Figure 6:**
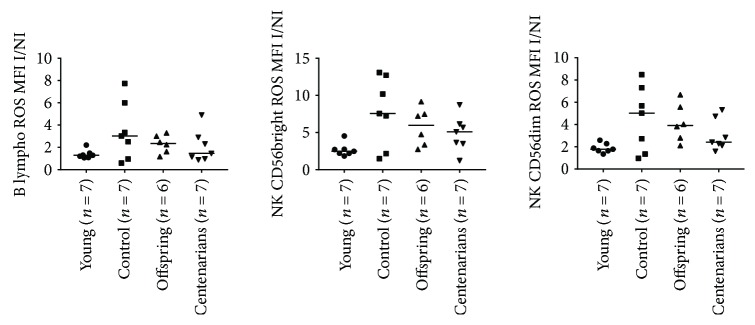
ANOVA for the ratios of the levels of ROS (expressed as MFI values of the ROS probe) between the I and NI conditions in B and NK subsets among the age groups. See Table 1 for overall ANOVA *p* values.

**Figure 7:**
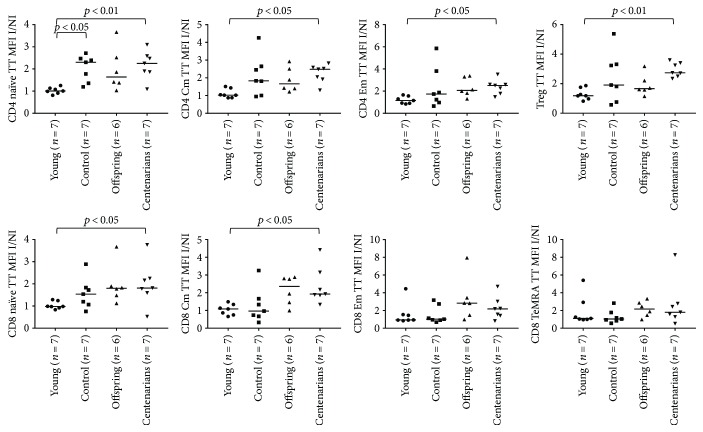
ANOVA for the ratios of the levels of GSH (expressed as MFI values of the TT probe) between the I and NI conditions in CD4 and CD8 subsets among the age groups. See Table 2 for overall ANOVA *p* values. Significant results of Dunn's posttest are illustrated on the plots.

**Figure 8:**
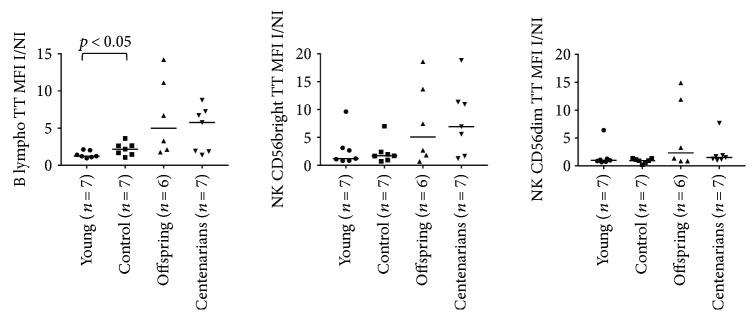
ANOVA for the ratios of the levels of GSH (expressed as MFI values of the TT probe) between the I and NI conditions in B and NK subsets among the age groups. See Table 2 for overall ANOVA *p* values. Significant results of Dunn's posttest are illustrated on the plots.

To investigate the hypothesis that a different response to induced oxidative stress can be detected among the age groups, the ratio of ROS and GSH MFI between the induced (I) and NI oxidative stress conditions in the PBMC subsets was calculated (see Tables S3 and S5 for ROS and GSH MFI values in I condition). ANOVA showed significant overall differences in the CD4 and CD8 naïve subsets (*p* = 0.0096 and *p* = 0.03, resp.) and a trend for the CD8 TeMRA and NK subsets (*p* = 0.0571 and *p* = 0.07 for both NK CD56bright/dim, resp.; see Figures 5 and 6 and Table 1). Higher ROS I/NI MFI ratios were observed for CTRL and OFF in almost all the T, B, and NK subsets in comparison with YO and CENT, although such differences were not statistically significant at an alpha level of 5% (only in CD4 naïve, ROS I/NI ratio was significantly lower in YO compared to CTRL and CENT; see Figures 5 and 6 and Table 1). When we analyzed the GSH I/NI MFI ratios, ANOVA showed significant overall differences in CD4/8 naïve, CD4/8 Cm, CD4 Em, Treg, and B lymph. In general, a trend for increasing median values was observed across the 4 groups in almost all subsets, CENT and OFF showing the highest values. Notably, in CD4/8 naïve, CD4/8 Cm, CD4 Em, and Tregs, Dunn's posttest showed a significant difference between YO and CENT, whereas significant differences between CTRL and YO were detected in CD4 naïve and B lymphocytes (see Figures 7 and 8 and Table 2).

### 3.4. Correlation between the Response to Induced Oxidative Stress and the Degree of Methylation of Oxidative Stress-Related Genes

**Figure 9:**
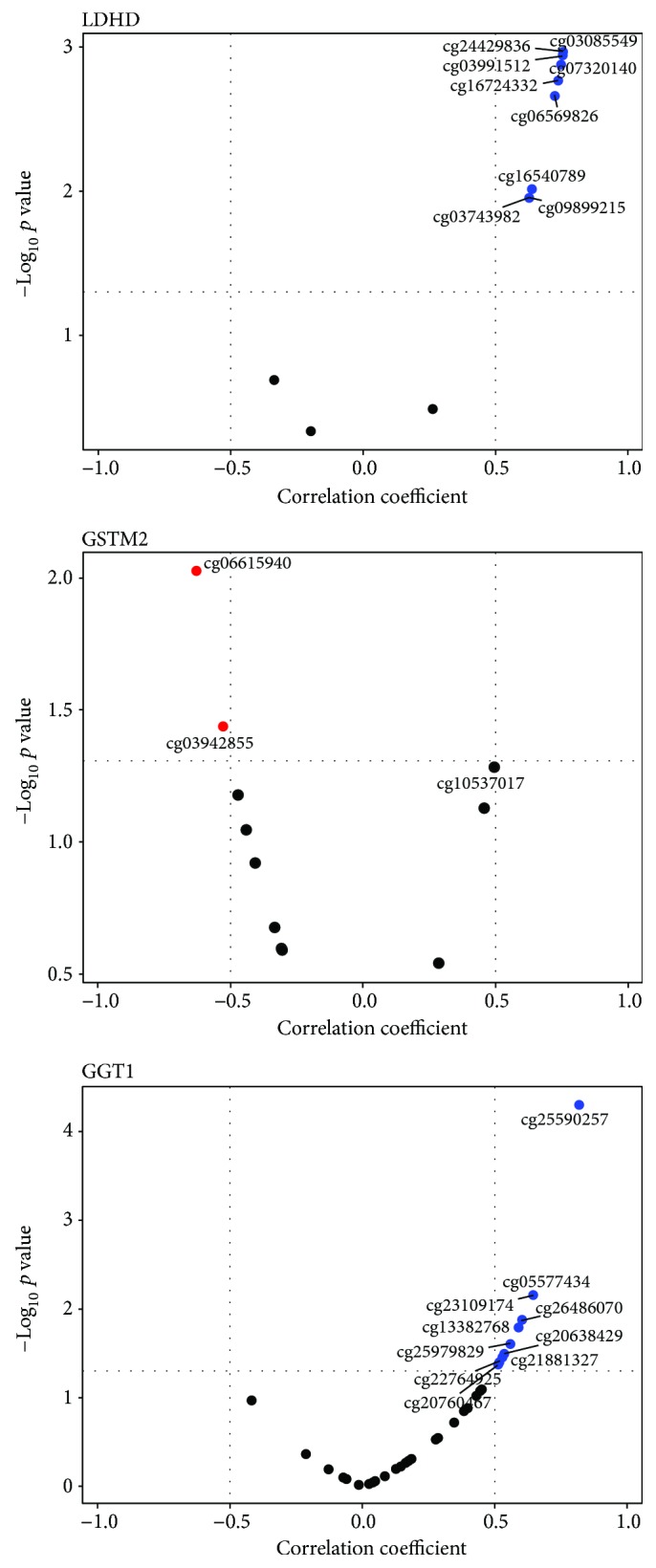
Analysis of correlation between the degree of methylation of the CpG sites and the response to induced oxidative stress (expressed as the ratio of MFI values of the ROS probe between I/NI conditions) for LDHD (top), GSTM2 (middle), and GGT1 (bottom) genes.

Finally, we evaluated if the differences in the response to induced oxidative stress described in the above paragraph could be related to epigenetic changes among the 4 groups. To this aim, we first considered the DNA methylation status of a list of genes involved in the response to oxidative stress (see Table S1) in a dataset including 47 CTRL, 63 OFF, and 82 CENT subjects. Several CpG sites showed significant DNA methylation differences in the three comparisons CENT versus CTRL, CENT versus OFF, and CTRL versus OFF (ANOVA *p* value < 0.05) (File S1). Several genes exhibited 2 or more adjacent differentially methylated CpG sites in the CENT versus CTRL and CENT versus OFF comparisons, suggesting a general epigenetic remodeling of these *loci* in the analyzed groups. These genes include GGT1, GGT6, GPX5, GPX6, GSTA 3-4, GSTM2, and LDHD (see Figures S1 and S2 sample plots). As a further step, we selected from the DNA methylation dataset the samples analyzed for the induced oxidative stress response (3 CTRL, 6 OFF, and 7 CENT) and correlated the degree of methylation of the abovementioned genes with the ROS I/NI ratio. Importantly, in this subcohort, the methylation profiles were comparable to those found in the whole cohort (see Figure S4 for an exemplative plot related to the GGT1 gene). As the methylation profile was assessed using PBMCs, we reanalyzed the ROS I/NI ratio gathering all the lymphocytes subsets together, including the monocyte population (percentage varying from 1 to 10% of the samples). The ROS I/NI ratio in CENT tended to be lower than in OFF and CTRL (Figure S3 and Table S6), confirming the above described results on separated cell populations, although the differences were not statistically significant according to ANOVA test. This was probably due to the small sample size of the CTRL group, as methylation data were available only for 3 subjects analyzed with flow cytometry. Notwithstanding, Spearman correlation between the ROS I/NI ratio and the DNA methylation resulted significant for several CpG sites within GGT1 and LDHD genes (positive correlation) and GSTM2 (negative correlation) (Figure 9).

## 4. Discussion

In this paper, by means of PFC, we assessed the differences in the response towards induced oxidative stress in the main subsets of PBMC among the age groups, enumerating at the same time the relative amounts of each cell subset, in order to describe the process of immunosenescence. Notably, we had the opportunity to collect and analyze PBMC samples from the centenarians' offspring. This unique feature allowed us to reveal the cell phenotype of subjects sharing with CENT possible variants of genes involved in the healthy ageing and longevity and to further compare such phenotype with the one of their age-matched CTRL. Our data in T cell compartment, both in CD4 and CD8, show an age-related decrease in the percentage of naïve T cells and an increase in almost all the memory T cell subsets, including Tregs. Although the number of subjects analyzed in these cohorts was limited (*n* = 27), the differences observed are highly significant and these data are in line with the literature. Taken together, our results consistently describe the process of thymic involution and memory inflation, especially in the effector compartment [31–33, 35, 36]. Notably, in almost all T cell subsets, the percentage of cells in OFF and CTRL was comparable, suggesting that the immunosenescence of the T cell compartment follows the common age-dependent pattern, rather than being correlated to the genetic background. Moreover, we found increased percentages of B cells from YO to OFF, whereas in CENT, the percentages were comparable to YO. This is in contrast with previous observations in humans, in which a decrease or no changes were observed [31, 34]. This could be related to the decrease in CD3 T cell percentages observed in the present study from YO to OFF, already described in literature [31], whereas in CENT, the percentage was similar to YO (data not shown). As the B and CD3 gates were drawn under the same parent gate (lymphocyte scatter), the decrease in CD3 affected the B cell percentage. In this case, absolute counts would have provided more information on the amount of B cells among the groups, but only if using whole blood with stain-lyse-no wash methods, a condition that was not applicable in the present study. Finally, we observed a decreased percentage of NK CD56dim cells in CTRL and OFF, whereas in CENT, the percentage of such cells was similar to YO. As already suggested, the preservation of the number and *bona fide* function of the NK cytotoxic subset could represent a hallmark of healthy ageing [31, 34]. Once the relative amount of each PBMC subset in the age groups was determined, the response of such subsets to the induced oxidative stress was investigated. Our results showed no appreciable differences in the relative amount of ROS at the baseline in the different PBMC subsets among the age groups. Our results at the baseline are different from those available in literature, showing in general an age-related increase of the cellular oxidative stress parameters [25–27]. This could be due to different factors including the cell type investigated, the analytical methodology used, and the source of biological material, that is, frozen PBMC. Indeed, this represents a limitation of the study, as it has already been shown that the freeze-thaw process can modify the signal from ROS [27]. However, the main objective of this study was to define the response to the dynamic changes in the oxidative stress in subjects of different ages, rather than a baseline. This could represent a better hallmark of healthy ageing and at the same time offer a more reliable measurement than the baseline, potentially biased by the freeze-thaw process. Thus, we compared the ratio between the MFI of ROS in I and NI conditions. The results showed that CENT resists better to the induced oxidative stress compared with OFF and YO in almost all the PBMC subsets, having a lower ratio, in many subsets comparable to YO. Although the post hoc comparisons failed to provide significant statistical differences intergroups (possibly due to the limited number of subjects analyzed and then to the biological variability), our data suggests that CENT may have adopted efficient strategies to counteract possible sudden changes in the redox environment. This can limit the oxidative damage and, thus, preserve a better cellular response especially in the immune system. Notably, these results are in line with those obtained by Alonso-Fernández et al. measuring superoxide anion production in stimulated (but also not stimulated) neutrophils in young, middle-aged, and centenarian subjects [29]. In a previous work, using different methodologies, an age-related decreased susceptibility to the oxidative stress-induced apoptosis in nonactivated PBMC was demonstrated [37]. It is tempting to speculate that if, on the one hand, the chronic exposure to ROS decreases the susceptibility to apoptosis leading to the accumulation of particular subsets, on the other hand, CENT may be better suited to counteract dynamic variations in oxidative stress, preserving a better cellular response in such subsets. In this context, several lines of research put efforts in defining genetic variants in oxidative stress response genes that can be associated with exceptional longevity [38], and even more interest relies around the epigenetic changes, such as DNA methylation, that can underline the ageing process and be hallmarks of healthy ageing [39–41]. Presently, and with the limited number of subjects analyzed, we showed for the first time a significant correlation between the response to the oxidative stress in the PBMC population and the degree of methylation in several CpG positions of selected genes involved in the response to oxidative stress (LDHD, GGT1, and GSTM2; [42–44]). These findings could have relevant functional implications, as the differential degree of methylation could control the mRNA expression [45] leading, in turn, to different protein levels [46]. Thus, at least from a hypothetical perspective, different amount of proteins could impact differently the oxidative stress response. For limitations due to the availability of the specific PBMC samples, we have not been able to test such hypothesis. Thus, additional experiments are needed, in order to ascertain whether differences in DNA methylation could be associated with variations in the gene (and protein) expression after dynamic changes in ROS level. In order to overcome possible limitations due to sample availability, PFC can be used for all the readouts, combining gene expression profiling with immunophenotyping and oxidative stress detection [47]. This would eventually allow a precise dissection of subset-related differences in the expression of selected genes, without the need to physically separate cells by cell sorting. When we approached the oxidative stress response from the antioxidant perspective, we observed an important decrease in GSH content at baseline in almost all subsets. An age-related decrease of GSH content has been already described in the lymphocyte population [26, 48], and the decline has been associated with a decrease in the *γ*-glutamil cysteine ligase (GCL), an important enzyme in the GSH biosynthesis, expression, and activity [49]. Notably, OFF showed the same GSH level as CENT, and much lower than the CTRL group. However, the induced oxidative stress promotes an increase in cellular GSH content [50] and, in our experimental model, we observed an age-related increase in GSH ratio after oxidative stress induction. In particular, CENT showed the highest fold-increase values in 6 out of 11 PBMC subsets analyzed, whereas OFF showed a GSH fold-increase value higher than CTRL in 8 out of 11 subsets. Strikingly, we observed a decrease in the baseline GSH content (ratio between I/NI condition < 1) after induction only in the YO and CTRL groups: in CD8 naïve, CD8Cm/Em, and in NK CD56dim subsets. This observation would suggest that CENT, consistent with the smaller fold-increase observed in ROS, promptly synthesizes and uses GSH to buffer the dynamic changes in oxidative stress level. Moreover, this could explain the fact that we did not observe drastic changes in the absolute fluorescence value of GSH. On the contrary, OFF produces GSH after oxidative stress induction but is not capable to efficiently use it to counteract the increase of cellular ROS. From a hypothetical point of view, we may speculate that CENT has a reduced capability to produce constitutive GSH but at the same time is more efficient in producing and using GSH under stress conditions. In contrast, YO and CTRL rely on their GSH stores to counteract the dynamic changes in the redox environment, whereas OFF promptly triggers GSH production but, unlike the CENT, appears not to be capable in using it to buffer the cellular ROS increase. Such observations, however, need to be confirmed in other independent cohorts of ageing subjects. Taken together, our results suggest that, at least in cultured PBMC, the resistance to changes in oxidative stress levels is a characteristic of the group of subjects who reached healthy ageing. In the immune system, this possibly implies that CENT could maintain good cell functions in order to efficiently fight infections. However, our observations must be placed into a more comprehensive framework as ageing involves complex processes shaped by genetic, epigenetic, and environmental factors. Thus, further investigations are needed to elucidate the mechanisms of the resistance to oxidative stress and to evaluate possible intervention (i.e., diet and lifestyle) to enhance such resistance in the ageing population.

## Figures and Tables

**Table 1:** Ratios of the MFI values of the ROS probe between the I and NI conditions in all the PBMC subsets among the age groups.

ROS I/NI ratio	YO	CTRL	OFF	CENT	Overall ANOVA *p* value
*CD4 naïve*	1.500	4.858	4.040	3.155	0.0096
*CD4 Cm*	1.631	2.724	3.256	2.992	0.2185
*CD4 Em*	2.423	2.279	3.336	1.622	0.1138
*Treg*	1.656	1.973	1.919	1.485	0.9240
*CD8 naïve*	1.247	3.432	3.798	3.281	0.0370
*CD8 Cm*	1.340	2.524	3.051	2.793	0.1657
*CD8 Em*	2.440	3.701	3.647	2.429	0.1745
*CD8 TeMRA*	2.225	3.129	3.585	1.953	0.0571
*B lymph*	1.289	3.023	2.346	1.457	0.2442
*NK CD56bright*	2.473	7.554	5.966	5.088	0.0727
*NK CD56dim*	1.782	5.021	3.915	2.410	0.0747

**Table 2:** Ratios of the MFI values of the GSH probe (Thiol Tracker, TT) between the I and NI conditions in all the PBMC subsets among the age groups.

GSH I/NI ratio	YO	CTRL	OFF	CENT	Overall ANOVA *p* value
*CD4 naïve*	1.004	2.301	1.638	2.253	0.0050
*CD4 Cm*	1.019	1.833	1.660	2.482	0.0246
*CD4 Em*	1.150	1.736	2.063	2.513	0.0194
*Treg*	1.180	1.906	1.661	2.737	0.0146
*CD8 naïve*	0.9818	1.538	1.803	1.811	0.0295
*CD8 Cm*	1.086	0.9634	2.357	1.928	0.0114
*CD8 Em*	0.9410	1.019	2.832	2.176	0.1413
*CD8 TeMRA*	1.105	1.038	2.166	1.794	0.2438
*B lymph*	1.234	2.173	4.990	5.755	0.0171
*NK CD56bright*	1.207	1.712	5.077	6.949	0.1504
*NK CD56dim*	0.9887	0.9486	2.321	1.513	0.0913

## Supplementary Materials

Supplementary Materials Table S1: list of the genes involved in response to the oxidative stress. Figure S1: percentage of methylation of the CpG sites throughout the GGT1 gene in the 3 ageing groups. Vertical bars represent the standard deviation of the mean of the methylation values for each CpG site in the 3 groups. Figure S2: percentage of methylation of the CpG sites throughout the GSTM2 gene in the 3 ageing groups. Vertical bars represent the standard deviation of the mean of the methylation values for each CpG site in the 3 groups. Figure S3: ratios of the MFI values of the ROS probe between the I and NI conditions in the whole PBMC population across 3 groups of ageing individuals (subcohort *n* = 16). Figure S4: percentage of methylation of the CpG sites throughout the GGT1 gene in the subcohort of 16 subjects. Vertical bars represent the standard deviation of the mean of the methylation values for each CpG site in the 3 groups. ANOVA between the three groups (CENT *n* = 7; OFF *n* = 6; CTRL *n* = 3) showed the following results: cg25590527: *p* = 0.0042; *q* = 0.1181; cg13825083: *p* = 0.0406; *q* = 0.1800; cg21881327: *p* = 0.019; *q* = 0.1788; cg09598276: *p* = 0.0304; *q* = 0.1788; cg01120527: *p* = 0.005; *q* = 0.1181; cg22123459: *p* = 0.003; *q* = 0.1181. Table S2: MFI values of the ROS probe in the NI condition among the age groups in the PBMC subsets. Table S3: MFI values of the ROS probe in the I condition among the age groups in the PBMC subsets. Table S4: MFI values of the GSH probe in the NI condition among the age groups in the PBMC subsets. Table S5: MFI values of the GSH probe in the I condition among the age groups in the PBMC subsets. Table S6: MFI values of the ROS and GSH probes in the NI, I, and I/NI conditions in the whole PBMC population among the age groups.
