# Supplementary material for: Evaluation of Lymphocyte Response to the Induced Oxidative Stress in a Cohort of Ageing Subjects, including Semisupercentenarians and Their Offspring
Source: Mediators Inflamm. 2018 Feb 19;2018:7109312. doi: 10.1155/2018/7109312 (PMC5842690; doi:10.1155/2018/7109312)
Supplement: Supplementary Materials — Table S1: list of the genes involved in response to the oxidative stress. Figure S1: percentage of methylation of the CpG sites throughout the GGT1 gene in the 3 ageing groups. Vertical bars represent the standard deviation of the mean of the methylation values for each CpG site in the 3 groups. Figure S2: percentage of methylation of the CpG sites throughout the GSTM2 gene in the 3 ageing groups. Vertical bars represent the standard deviation of the mean of the methylation values for each CpG site in the 3 groups. Figure S3: ratios of the MFI values of the ROS probe between the I and NI conditions in the whole PBMC population across 3 groups of ageing individuals (subcohort n = 16). Figure S4: percentage of methylation of the CpG sites throughout the GGT1 gene in the subcohort of 16 subjects. Vertical bars represent the standard deviation of the mean of the methylation values for each CpG site in the 3 groups. ANOVA between the three groups (CENT n = 7; OFF n = 6; CTRL n = 3) showed the following results: cg25590527: p = 0.0042; q = 0.1181; cg13825083: p = 0.0406; q = 0.1800; cg21881327: p = 0.019; q = 0.1788; cg09598276: p = 0.0304; q = 0.1788; cg01120527: p = 0.005; q = 0.1181; cg22123459: p = 0.003; q = 0.1181. Table S2: MFI values of the ROS probe in the NI condition among the age groups in the PBMC subsets. Table S3: MFI values of the ROS probe in the I condition among the age groups in the PBMC subsets. Table S4: MFI values of the GSH probe in the NI condition among the age groups in the PBMC subsets. Table S5: MFI values of the GSH probe in the I condition among the age groups in the PBMC subsets. Table S6: MFI values of the ROS and GSH probes in the NI, I, and I/NI conditions in the whole PBMC population among the age groups. [file 7109312.f1.pdf]

Gene	Description
CAT	Catalase; Converts H202 in H2O and O2
GCLC	Glutamate-Cysteine Ligase Catalytic Subunit; glutathione synthesis
GCLM	Glutamate-Cysteine Ligase Modifier Subunit; glutathione synthesis
GGCT	Gamma-Glutamylcyclotransferase; gluthatione catabolism
GGT1	Gamma-Glutamyltransferase 1; glutathione catabolism
GGT5	Gamma-Glutamyltransferase 5; glutathione catabolism
GGT6	Gamma-Glutamyltransferase 6; glutathione catabolism
GGT7	Gamma-Glutamyltransferase 7; glutathione catabolism
GPX1	Glutathione Peroxidase 1; reduction of H202 in H20 using glutathione (ubiquitary)
GPX2	Glutathione Peroxidase 2; reduction of H202 in H20 (gastrointestinal)
GPX3	Glutathione Peroxidase 3; reduction of H202 in H20 (plasma)
GPX4	Glutathione Peroxidase 4; reduction of H202 in H20 (phospholipid hydroperoxidase)
GPX5	Glutathione Peroxidase 5; reduction of H202 in H20 (Epydidimis)
GPX6	Glutathione Peroxidase 6; reduction of H202 in H20 (Olfactory)
GPX7	Glutathione Peroxidase 7; reduction of H202 in H20 (Ephitelium gastroesophagean)
GSR	Glutathione S-reductase; reduces oxidized glutahione
GSS	Glutathione Synthetase; glutathione synthesis
GSTA1	Glutathione S-transferase Alpha 1; add reduced glutathione to electrophilic compounds
GSTA2	Glutathione S-transferase Alpha 2
GSTA3	Glutathione S-transferase Alpha 3
GSTA4	Glutathione S-transferase Alpha 4
GSTA5	Glutathione S-transferase Alpha 5
GSTK1	Glutathione S-transferase K1; peroxisomal

GSTM1	Glutathione S-transferase Mu 1; add reduced glutathione to electrophilic compounds
GSTM2	Glutathione S-transferase Mu 2
GSTM3	Glutathione S-transferase Mu 3
GSTM4	Glutathione S-transferase Mu 4
GSTM5	Glutathione S-transferase Mu 5
GSTO1	Glutathione S-transferase Omega 1; thiol transferase and dehydroascorbate reductase
GSTO2	Glutathione S-transferase Omega 2
GSTP1	Glutathione S-transferase Pi 1; add reduced glutathione to electrophilic compounds
GSTT1	Glutathione S-transferase Theta 1; haplotype specific
GSTT2B	Glutathione S-transferase Theta 1; pseudogene
LAP3	Leucine aminopeptidase 3; glutathione metabolism
LDHD	D-Lactate dehydrogenase; methylglyoxal degradation
MGST1	Microsomal Glutathione S-transferase 1; production of leukotrienes
MGST2	Microsomal Glutathione S-transferase 2
MGST3	Microsomal Glutathione S-transferase 3
NOX3	NADPH Oxidase 3; generation of ROS in inner ear cells
NOX4	NADPH Oxidase 4; generation of ROS in non phagocytic cells
NOX5	Calcium dependent-NADPH Oxidase 4; generation of ROS in testis and lymph nodes
OPLAH	5-Oxoprolinase; Glutathione metabolism
SOD1	Superoxide Dismutase 1, Soluble; converts superoxide anion to H2O2 and O2
SOD2	Superoxide Dismutase 2, Mitochondrial; converts superoxide anion to H2O2 and O2
SOD3	Superoxide Dismutase 3, Extracellular; converts superoxide anion to H2O2 and O2
TXN	Thioredoxin; response to intracellular nitric oxide
TXN2	Thioredoxin 2; regulation of membrane potential and oxidant.induced apoptosis

TXNRD1	Thioredoxin reductase 1
TXNRD2	Thioredoxin reductase 2

Suppl. Tab 1. List of the genes involved in the response to the oxidative stress.

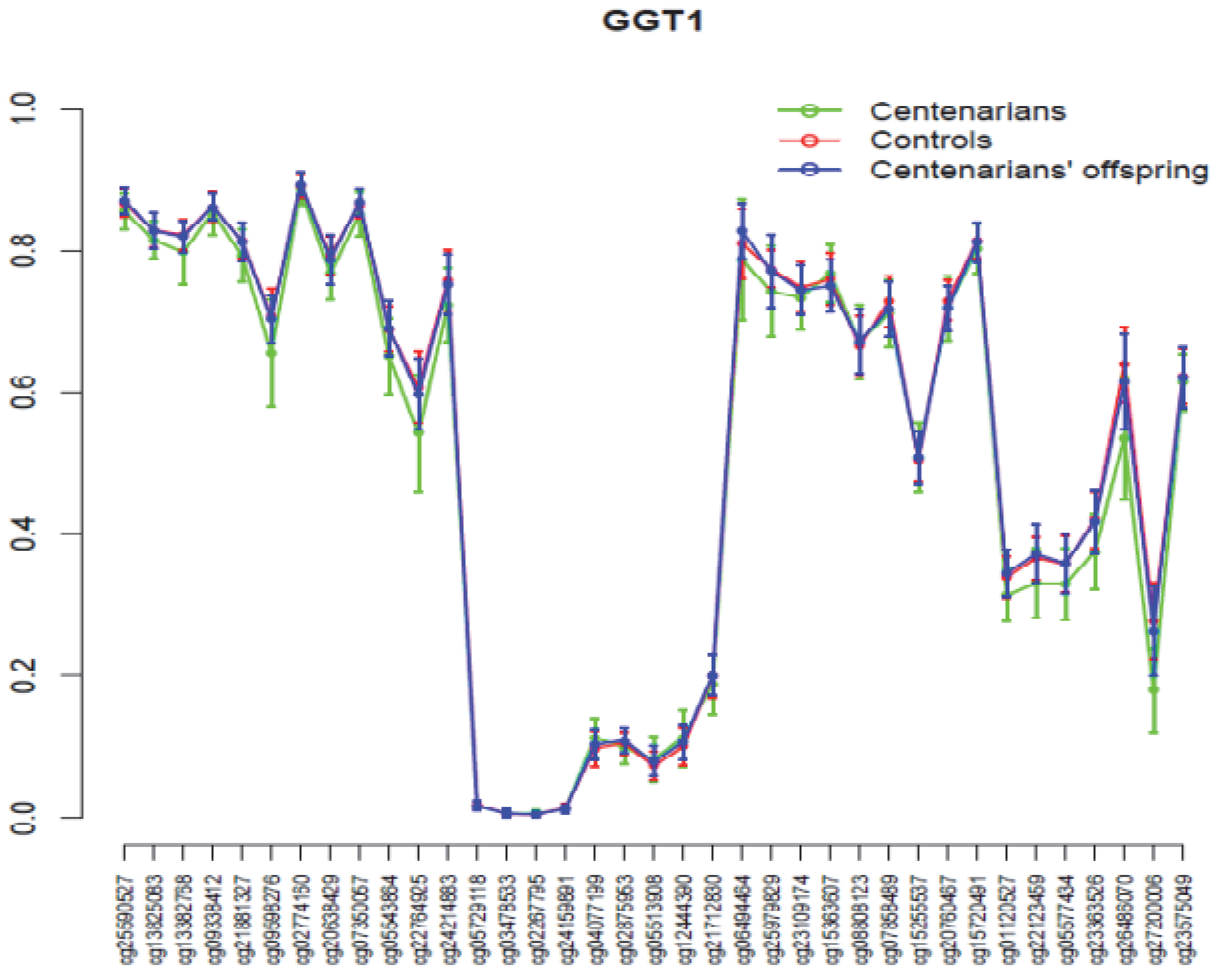
GGT1
1.0
0.8
0.6
0.4
0.2
0.0
Centenarians
Controls
Centenarians' offspring
cg25590527
cg13825083
cg13382758
cg09338412
cg21881327
cg09698276
cg02774150
cg20638429
cg07350057
cg05543864
cg22764925
cg24214883
cg05729118
cg03478533
cg02267795
cg24159891
cg04077199
cg02875963
cg05513308
cg12444390
cg21712830
cg06494464
cg25973829
cg23109174
cg15363607
cg08808123
cg07858489
cg15255537
cg20760467
cg15720491
cg01120527
cg22123459
cg05577434
cg23363526
cg26486070
cg27200006
cg23575049

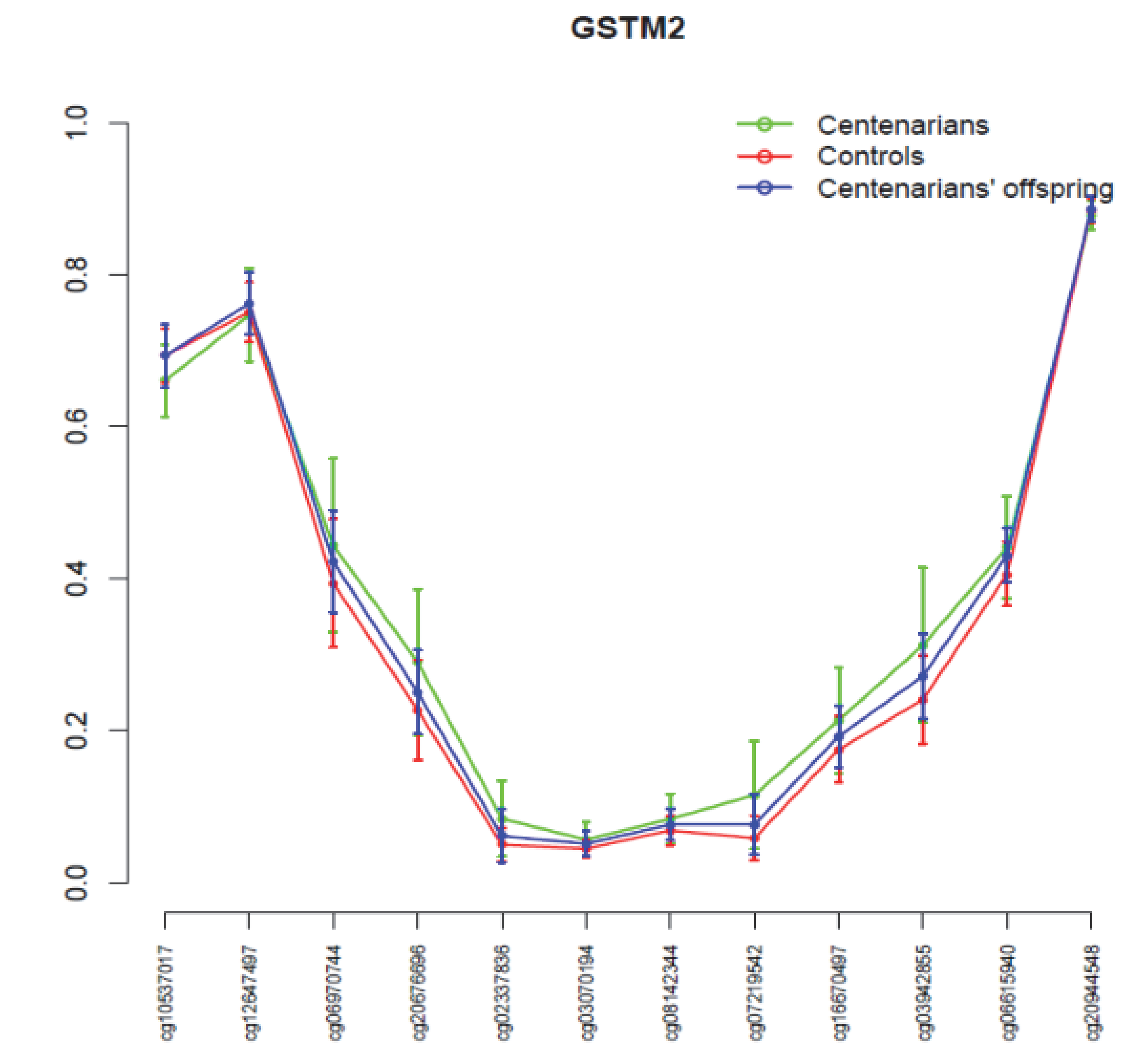
GSTM2
Centenarians
Controls
Centenarians' offspring
1.0
0.8
0.6
0.4
0.2
0.0
cg10537017
cg12647497
cg06970744
cg20676696
cg02337836
cg03070194
cg08142344
cg07219542
cg16670497
cg03942855
cg06615940
cg20944548

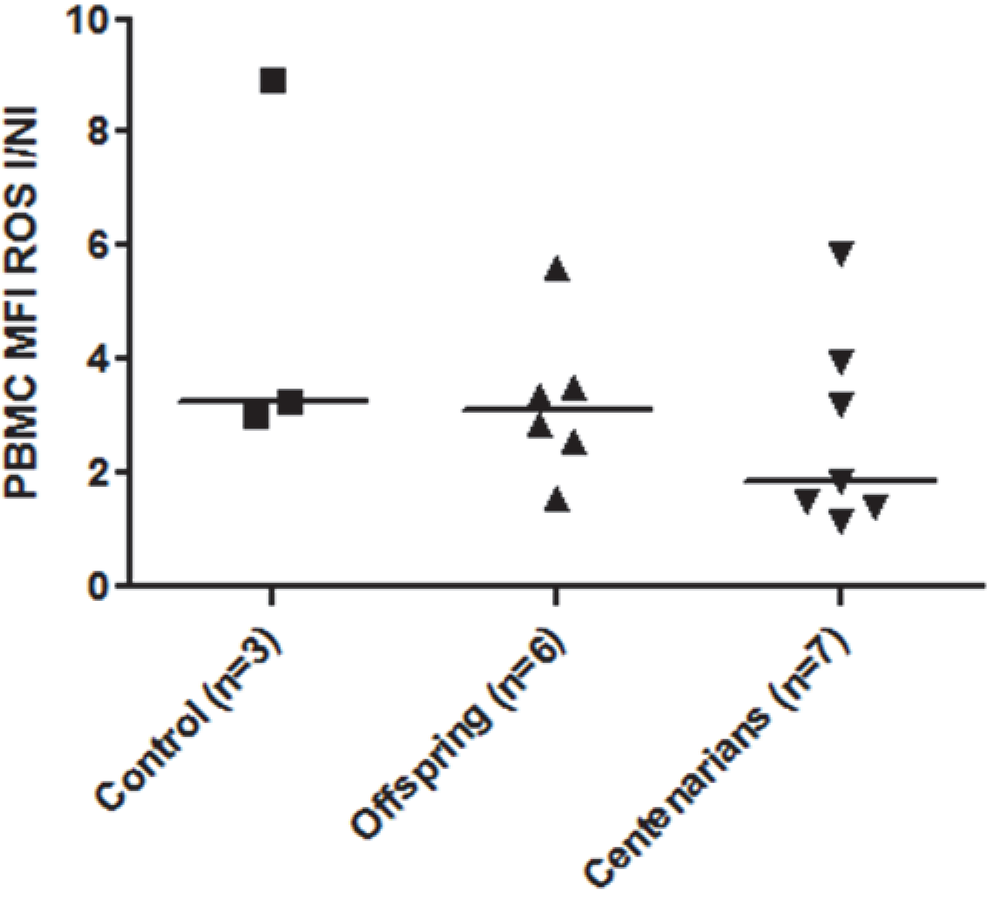

PBMC MFI ROS I/NI
10
8
6
4
2
0
Control (n=3)
Offspring (n=6)
Centenarians (n=7)

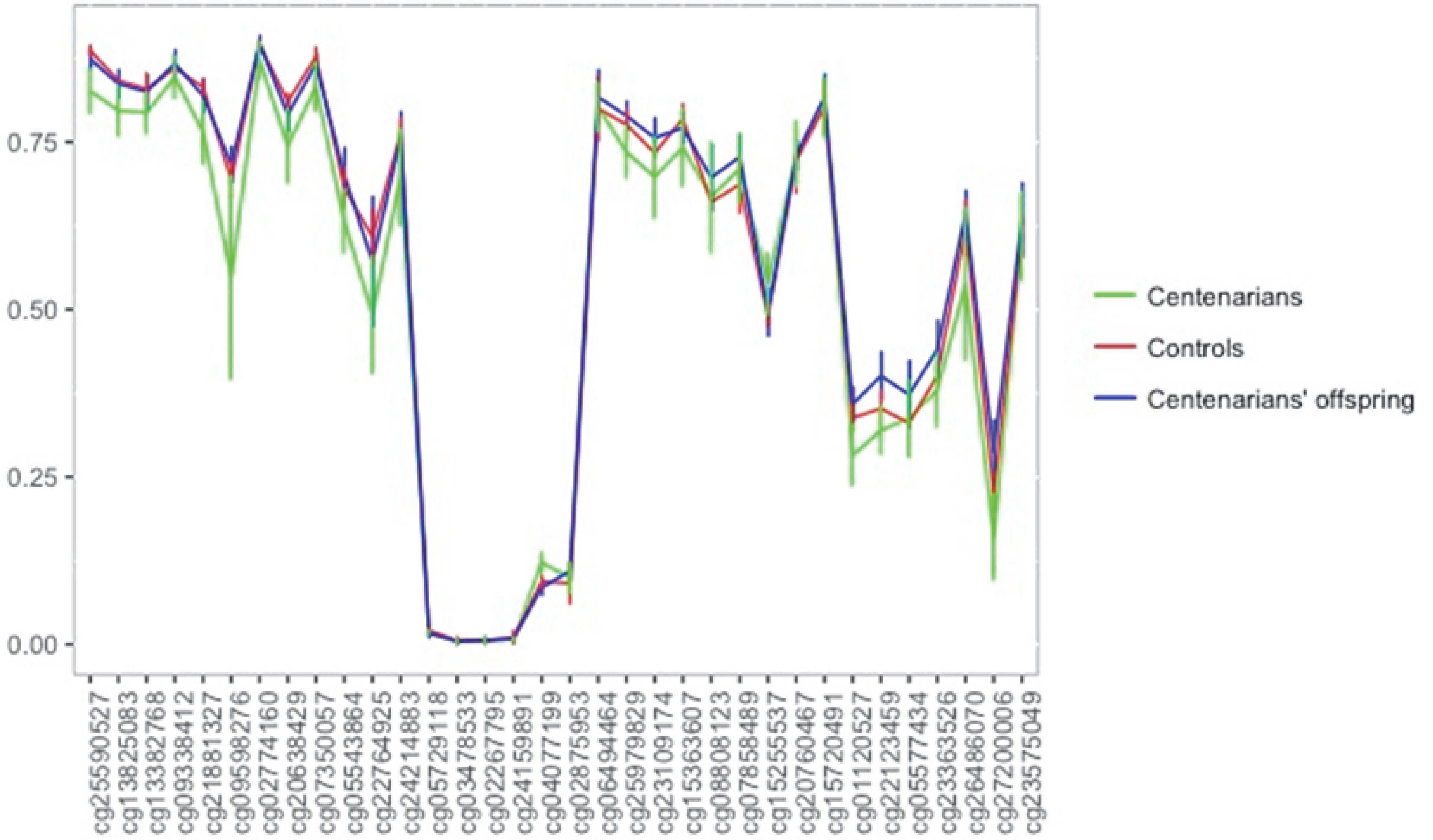

0.75
0.50
0.25
0.00
cg25590527
cg13825083
cg13382768
cg09338412
cg21881327
cg09598276
cg02774160
cg20638429
cg07350057
cg05543864
cg22764925
cg24214883
cg05729118
cg03478533
cg02267795
cg24159891
cg04077199
cg02875953
cg06494464
cg25979829
cg23109174
cg15363607
cg08808123
cg07858489
cg15255537
cg20760467
cg15720491
cg01120527
cg22123459
cg05577434
cg23363526
cg26486070
cg27200006
cg23575049
Centenarians
Controls
Centenarians' offspring

ROS MFI NI	YO	CTRL	OFF	CENT	Overall ANOVA p-value
CD4 Naive	1343	734.2	1007	974.5	0.3503
CD4 Cm	1249	718.6	957.4	886.9	0.5464
CD4 Em	578.5	499.3	578.9	582.8	0.6076
TReg	1548	846.2	1143	1083	0.5581
CD8 Naive	899.4	453.5	681.4	666.2	0.4657
CD8 Cm	990.5	439.5	842.0	860.6	0.4540
CD8 Em	418.9	316.6	412.3	428.9	0.4498
CD8 TemRA	539.9	360.9	425.6	385.2	0.1695
B Lymph	950.5	443.1	781.6	957.0	0.5969
NK CD56 bright	455.4	154.9	280.9	309.4	0.3714
NK CD56 dim	460.7	235.8	374.2	366.8	0.5074

Tab. S2: MFI values of the ROS probe in the NI condition among the age groups in the PBMC subsets.

ROS MFI I	YO	CTRL	OFF	CENT	Overall ANOVA p-value
CD4 Naive	1490	4044	4038	3734	0.0966
CD4 Cm	1950	2547	3075	3672	0.4820
CD4 Em	1401	1544	1859	1449	0.5285
TReg	1445	1762	2095	2072	0.6754
CD8 Naive	1086	1661	2385	1902	0.0677
CD8 Cm	1327	1644	2220	1989	0.3397
CD8 Em	1288	1246	1497	1042	0.4705
CD8 TemRA	1227	1516	1670	1026	0.3013
B Lymph	1214	1339	1788	1327	0.4530
NK CD56 bright	1214	1374	1610	1080	0.8157
NK CD56 dim	939.5	1214	1285	999.2	0.5092

Tab. S3: MFI values of the ROS probe in the I condition among the age groups in the PBMC subsets.

TT MFI NI	YO	CTRL	OFF	CENT	Overall ANOVA p-value
CD4 Naive	11915	6474	1056	1190	0.1301
CD4 Cm	13667	7139	1245	1212	0.1631
CD4 Em	12402	6830	1361	1207	0.2005
TReg	9717	5321	1140	1015	0.1497
CD8 Naive	13742	7686	1644	3033	0.3759
CD8 Cm	15390	9705	1617	1691	0.2287
CD8 Em	15984	12797	1748	1882	0.2805
CD8 TemRA	14521	13042	1923	2988	0.5022
B Lymph	11440	9719	1632	891.5	0.5020
NK CD56 bright	15092	15365	1851	1771	0.3548
NK CD56 dim	12992	28325	4693	570.3	0.0784

Tab. S4: MFI values of the GSH probe in the NI condition among the age groups in the PBMC subsets.

TT MFI I	YO	CTRL	OFF	CENT	Overall ANOVA p-value
CD4 Naive	10828	12544	2770	2216	0.5349
CD4 Cm	12132	13087	2851	2479	0.6001
CD4 Em	13464	11890	3875	3035	0.7438
TReg	9217	8543	1572	2755	0.8248
CD8 Naive	12119	12464	3098	1587	0.8631
CD8 Cm	13579	10018	3998	2252	0.7331
CD8 Em	14290	12245	5651	1675	0.6108
CD8 TemRA	15394	13057	4169	2789	0.5856
B Lymph	12757	19274	8419	5986	0.3289
NK CD56 bright	18212	29518	16884	19388	0.3811
NK CD56 dim	10694	30010	10029	2635	0.5513

Tab. S5: MFI values of the GSH probe in the I condition among the age groups in the PBMC subsets.

	CTRL	OFF	CENT	Overall ANOVA p-value
PBMC ROS NI	380.6	732.2	597	0.1601
PBMC ROS I	1895	1931	1223	0.3449
PBMC ROS I/NI	3.278	3.107	1.841	0.4040
PBMC TT NI	10056	1502	1658	0.7061
PBMC TT I	13972	3247	1917	0.7714
PBMC TT I/NI	1.840	1.999	1.822	0.6424

Suppl. Tab.6 MFI values of the ROS and GSH probes in the NI, I and I/NI conditions in the whole PBMC population among the age-groups.
